# Curcumin Modulates Platelet Activation and ROS Production Induced by Amyloid Peptides: New Perspectives in Attenuating Prothrombotic Risk in Alzheimer’s Disease Patients

**DOI:** 10.3390/nu16244419

**Published:** 2024-12-23

**Authors:** Serena Rustichelli, Cristina Lanni, Marta Zarà, Gianni Francesco Guidetti, Mauro Torti, Ilaria Canobbio

**Affiliations:** 1 University School for Advanced Studies (IUSS), 27100 Pavia, Italy; serena.rustichelli@iusspavia.it; 2 Department of Biology and Biotechnology, University of Pavia, 27100 Pavia, Italy; marta.zara@unipv.it (M.Z.); gianni.guidetti@unipv.it (G.F.G.); mauro.torti@unipv.it (M.T.); 3 Department of Drug Sciences, University of Pavia, 27100 Pavia, Italy; cristina.lanni@unipv.it

**Keywords:** curcumin, amyloid peptides, platelet activation, reactive oxygen species

## Abstract

Background/Objectives: Amyloid peptides, whose accumulation in the brain as senile plaques is associated with the onset of Alzheimer’s disease, are also found in cerebral vessels and in circulation. In the bloodstream, amyloid peptides promote platelet adhesion, activation, oxidative stress, and thrombosis, contributing to the cardiovascular complications observed in Alzheimer’s disease patients. Natural compounds, such as curcumin, are known to modulate platelet activation induced by the hemostatic stimuli thrombin and convulxin. In this study, we investigated the ability of curcumin to modulate platelet activation triggered by amyloid peptides, and we compared its effects with those displayed on platelet activation induced by physiological agonists. Methods: Commercial ultrapure curcumin was used, and platelet aggregation, granule secretion, phosphorylation of selected signaling proteins, and reactive oxygen species production were analyzed on isolated human platelets. Results: Our results demonstrate that curcumin effectively suppressed platelet aggregation induced by fibrillar amyloid peptides. This effect was associated with the reduction in intracellular signaling pathways involving PKC, PI3K, and MAPK. By contrast, platelet aggregation and activation induced by thrombin and convulxin were only partially reduced by preincubation with curcumin. Moreover, curcumin completely suppressed granule secretion only when platelets were stimulated with hemostatic agonists, but it had no effects upon stimulation with amyloid peptides. Additionally, curcumin reduced the production of reactive oxygen species induced by amyloid peptides with a stronger efficiency compared to platelets stimulated with thrombin. Conclusions: These results indicate that curcumin displays selective and potent inhibitory activity on platelet responses to pathological stimuli, such as fibrillar amyloid peptides.

## 1. Introduction

Alzheimer’s disease (AD) is the most invalidating dementia in the elderly and is correlated with the abnormal accumulation of heterogeneous Aβ peptides in the brain parenchyma, in the form of senile plaques. Aβ peptides are also found in the circulation, where they accumulate in cerebral vessels, leading to cerebral amyloid angiopathy [1]. Amyloid peptides found in plasma are mostly derived from platelets [2], as platelets contain high amounts of the amyloid precursor protein APP, as well as all of the necessary secretases to generate amyloid peptides through the amyloidogenic pathway [3]. Platelets are primary involved in hemostasis and thrombosis, but evidence suggests that they may also contribute to the progression of AD by releasing Aβ and, thus, by increasing the pool of circulating amyloid peptides, with pro-inflammatory and pro-thrombotic activities. We and others have demonstrated that platelet-derived amyloid peptides activate platelets [4,5,6,7], promote reactive oxygen species (ROS) formation [8], and contribute to thrombosis [9]. As a result, AD patients are at higher risk of developing cardiovascular disease (CVD).

The possibility of modulating intracellular signaling pathways for platelet activation to mitigate thrombosis in CVD and AD is an important goal for the improvement of quality of life in patients. Various natural compounds, such as curcumin, are currently under investigation as potential platelet inhibitors. Curcumin is a bioactive molecule derived from the *Curcuma longa* L. (turmeric) rhizome, widely used in Indian cuisine, medicine, and as an ingredient in nutraceutics. Several studies have indicated that curcumin possesses potent antioxidant, immunomodulatory, and anti-inflammatory properties, with cardio- and neuroprotective effects [10]. Curcumin also possesses anti-platelet activity. Curcumin and curcuminoids affect thrombin-induced platelet activation and aggregation by inhibiting platelet adhesion, P-selectin, and integrin αIIbβ3 expression [11]. Curcumin inhibits MAPK, Akt, and Src family kinases and signaling proteins involved in platelet activation induced by thrombin, ADP, collagen, and convulxin [12,13], increases the phosphorylation of VASP by protein kinase A downstream of adenosine A2A receptor activation [14], and blocks the formation of TxA2 through the inhibition of cytosolic phospholipase A2 and COX-1 activity [15,16,17]. Curcumin displays antioxidant activities [18] and reduces superoxide anion formation associated with platelet aggregation induced by arachidonic acid and ADP [17]. Curcumin also has anti-amyloidogenic effects and inhibits the formation of large toxic Aβ oligomers, thus limiting the formation of insoluble amyloid fibrils [19]. Therefore, curcumin is also suitably placed to potentially affect platelet activation by amyloid peptides in the progression of AD. However, this possibility has never been investigated.

In the present study, we analyzed the ability of curcumin to modulate platelet activation and ROS formation induced by amyloid peptides, and we compared this effect to that elicited by curcumin on platelets’ responsiveness to classical hemostatic agonists such as thrombin and convulxin. Our results demonstrate that the inhibitory effects of curcumin are more potent and selective upon platelet stimulation with amyloid peptides compared to classical physiological agonists, thus supporting the possible use of curcumin in the treatment of CVD in AD patients.

## 2. Materials and Methods

### 2.1. Materials

Amyloid peptides were synthesized by Lifetein (Somerset, NJ, USA). The sequences of the peptides were as follows:

Aβ40 (DAEFRHDSGYEVHHQKLVFFAEDVGSNKGAIIGLMVGGVV).

Aβ42 (DAEFRHDSGYEVHHQKLVFFAEDVGSNKGAIIGLMVGGVVIA).

The redox-sensitive fluorescent dye 2′,7′-dichlorodihydrofluorescein diacetate (H2DCFDA) was obtained from Calbiochem (Sigma-Aldrich, St. Louis, MO, USA). Apyrase I and VII, prostaglandin E1, U46619, thrombin, DMSO, HEPES, and the ultrapure curcumin (#08511) were obtained from Sigma-Aldrich (St. Louis, MO, USA). Bovine serum albumin was obtained from SERVA Electrophoresis (Heidelberg, Germany). Convulxin was a generous gift from Professor K.J. Clementson (Bern, Switzerland). Chemiluminescent ECL substrate was obtained from Millipore (Burlington, MA, USA). Reagents and instruments for SDS-PAGE and Western blotting were obtained from Sigma-Aldrich and Bio-Rad (Hercules, CA, USA). The monoclonal antibody anti-tubulin (sc-32293) was obtained from Santa Cruz Biotechnology (Dallas, TX, USA). Anti-pleckstrin (ab-17020) was obtained from Abcam (Cambridge, UK). Anti-phospho-Akt (S473) (#9275), anti-phospho-GSK3α/β (S21/9) (#9331), anti-phospho-PKC substrates (#2261), anti-phospho-ERK1/2 (T202/Y204) (#9101), and anti-phospho-p38 MAPK (T180/Y182) (#9211) were obtained from Cell Signaling Technology (Danvers, MA, USA). The secondary antibodies peroxidase-conjugated anti-mouse and anti-rabbit were obtained from Sigma-Aldrich.

### 2.2. Preparation of Fibrillar Amyloid Peptides

Synthetic amyloid peptides Aβ40, Aβ42, and respective scrambled peptides were purchased in lyophilized form, dissolved in 5 mM DMSO, and stored at −20 °C until use. Peptide fibrillation was performed essentially as described in [10] and initiated by diluting amyloid peptides in phosphate-buffered saline (PBS, pH 7.4) at a final concentration of 0.25 mmol/L at 37 °C for 24 h. Fibrillation was monitored using Thioflavin T (ThT) and Congo Red staining [8].

### 2.3. Preparation of Human Platelets

Washed human platelets were obtained from fresh buffy-coat bags, collected from healthy donors as previously described [8]. All volunteers provided written informed consent in accordance with the Declaration of Helsinki. Briefly, the buffy coat was diluted by one-third using a 1:10 solution of ACD (152 mM sodium citrate, 130 mM citric acid, and 112 mM glucose) and Tyrode’s buffer (10 mM HEPES, 137 mM NaCl, 2.9 mM KCl, 12 mM NaHCO_3_, pH 7.4) and then centrifuged at 120× *g* for 10 min at room temperature. Platelet-rich plasma was collected, and 0.2 U/mL apyrase grade I and 1 μM prostaglandin E1 were added. Platelets were separated by centrifugation at 720× *g* for 15 min and then washed with PIPES buffer (20 mM PIPES, 137 mM NaCl, pH 6.5). Finally, the platelets were resuspended in Tyrode’s buffer (10 mM HEPES, 137 mM NaCl, 2.9 mM KCl, 12 mM NaHCO_3_, 5.5 mM glucose, 1 mM CaCl_2_, 2 mM MgCl_2_,) containing 0.1% BSA. The cell count was typically adjusted to 1 × 10^9^ platelets/mL.

### 2.4. Platelet Aggregation Assay

Aggregation of platelets was evaluated in a light transmission aggregometer (Chrono-Log Corporation, Havertown, PA, USA) and monitored continuously for up to 5 min, using the AGGRO/LINK software (version 5.2). All of the experiments were performed with 0.3 mL samples of washed platelets at a concentration of 3 × 10^8^ platelets/mL, in the presence of 1 mM CaCl_2_, 5.5 mM glucose, and 0.5 mM MgCl_2_, and maintained at 37 °C for 30 min. The platelets were preincubated with curcumin (dissolved in DMSO) or vehicle, specific inhibitors, or receptor antagonists for 10 min and then stimulated with fibrillar amyloid peptides, thrombin, U46619, and convulxin, as indicated, under constant stirring at 37 °C.

### 2.5. SDS-PAGE, Western Blotting, and Immunoblotting

For the analysis of protein phosphorylation, platelet samples (1 × 10^9^ platelets/mL, 0.2 mL) were lysed with SDS sample buffer 3X (37.5 mM TRIS, 288 mM glycine, 6% SDS, 1.5% DTT, 30% glycerol, 0.03% bromophenol blue, and 3% 2-mercaptoethanol, pH 8.3) and boiled for 3 min at 96 °C. Aliquots of platelet lysates (20 µL) were separated by SDS-PAGE and then transferred onto PVDF membranes. The membranes were blocked for 1 h with 5% BSA in TBS (20 mM Tris/HCl, pH 7.5, 0.5 mM NaCl) and incubated overnight at 4 °C under gentle agitation. Immunoblotting analysis was performed using the following antibodies and dilutions: anti-phospho-GSK3α/β (S21/9), anti-phospho-Akt (S473), anti-phospho ERK1/2 (T202/Y204), anti-phospho p38MAPK (T180/Y182), anti-tubulin, anti-pleckstrin (1:1000), and anti-PKC phospho-substrates (1:2000).

The membranes were then extensively washed with 0.1% Tween 20 in TBS and incubated with peroxidase conjugated with the appropriate HRP-conjugated secondary antibodies for 1 h. Anti-mouse and anti-goat were diluted 1:5000, while anti-rabbit was diluted 1:2000. Proteins were visualized with a chemiluminescence reaction after repeated washing. Imaged were acquired with the ChemiDoc XRS system (Bio-Rad). The densitometric intensities of the bands were determined by using Quantity One-4.6.8 software (Bio-Rad).

### 2.6. Flow Cytometric Analysis of Platelet Surface Expression of CD62P

Samples of 1 × 10^8^ washed platelets, in the presence of 1 mM CaCl_2_, were untreated or preincubated with curcumin for 10 min at 37 °C without stirring. The platelets were activated by agonists for 5 min in the dark, and the reaction was stopped by adding 1% paraformaldehyde. The platelet samples were then labeled with BV421-conjugated anti-human CD62P antibody (P-selectin). Platelet activation was evaluated as the percentage of P-selectin expressed on the platelet surface by using a BD FACSLyric instrument (BD Biosciences, San Diego, CA, USA) at the PASS-BioMed facility of the University of Pavia (Lombardia, Italy).

### 2.7. Measurement of ROS Production

Samples of 1 × 10^8^ washed platelets were preloaded with 10 µM H2DCF-DA, a fluorescent probe for ROS detection, for 20 min at 37 °C. Following the incubation, 1 mM CaCl_2_ was added to platelets. The samples were untreated or preincubated with curcumin for 10 min and then stimulated with thrombin and convulxin for 5 min, with Aβ40, Aβ42, or the corresponding scrambled peptide for 15 min, and then diluted 10-fold in calcium-free Tyrode’s buffer containing 0.1% BSA and 10 µM H2DCFDA, before being analyzed immediately with a BD FACSLyric instrument.

### 2.8. Statistical Analysis

Data are presented as the mean ± SEM. Statistical analysis was performed using GraphPad Prism software, version 8.3.0, and statistical significance was analyzed using Student’s *t*-test for comparisons between two groups; * *p* < 0.05; ** *p* < 0.01; *** *p* < 0.001.

## 3. Results

### 3.1. Curcumin Is More Potent in Reducing Platelet Aggregation Induced by Amyloid Peptides Compared to Classical Agonists

**Figure 1:**
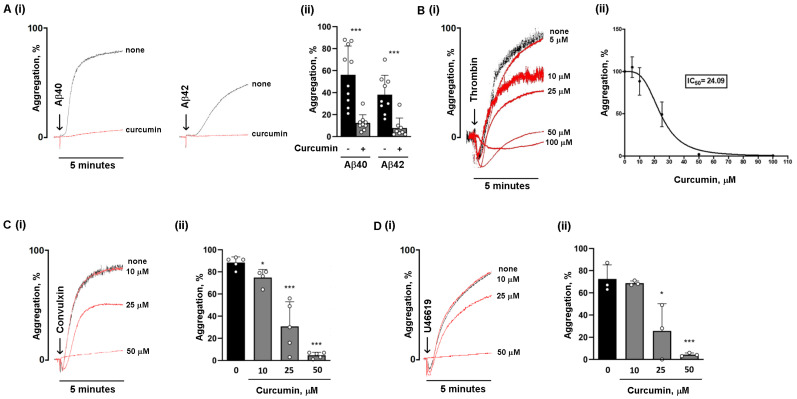
Curcumin reduces platelet aggregation induced by amyloid peptides and hemostatic agonists: (**A**) (**i**) Washed human platelets (3 × 10^8^ platelets/mL) were preincubated with 25 µM curcumin (red curves) or vehicle (black curve) for 10 min, stimulated with 10 µM fibrillar amyloid peptides Aβ40 and Aβ42 under constant stirring, and their aggregation was monitored. (**ii**) The histogram shows the mean ± SEM of the % of maximal aggregation in 8 to 10 independent experiments. (**B**) (**i**) Washed human platelets were preincubated with increasing concentrations of curcumin (5 to 100 µM) (red curves) or vehicle (black curve) for 10 min at 37 °C and stimulated with 0.04 U/mL thrombin; the respective IC_50_ is shown in (**ii**). Washed human platelets (3 × 10^8^ platelets/mL) were preincubated with increased concentrations of curcumin (10 to 50 µM) (red curves) or vehicle (black curve) for 10 min and stimulated with (**C**) 100 ng/mL convulxin and (**D**) 0.5 mM U46619, and aggregation was monitored for 5 min. Representative curves are reported in (**i**). The quantification of maximal aggregation is shown in (**ii**), as the mean ± SEM of 4 to 5 different experiments; * *p* < 0.05, *** *p* < 0.001.

Activated platelets released the amyloid peptides Aβ40 and Aβ42 [20]. Amyloid peptides are platelet agonists and promote platelet adhesion, activation, and aggregation in their monomeric, oligomeric, and fibrillar forms [5,8,21]. In this study, fibrillar amyloid peptides Aβ40 and Aβ42, obtained as described in [8], were used to stimulate platelet aggregation in the presence or absence of curcumin. Washed human platelets were preincubated with 25 µM curcumin for 10 min at 37 °C, and then stimulated with 10 µM fibrillar Aβ40 and Aβ42. Platelet aggregation was monitored by light transmission aggregometry. We confirmed that fibrillar Aβ40 is a more potent inducer of platelet aggregation than Aβ42 (maximal aggregation %: Aβ40: 60.1 ± 8.14 versus Aβ42: 41.0 ± 5.79). For both Aβ40 and Aβ42, corresponding scrambled peptides used as controls were not able to induce any platelet aggregation [8]. Platelet preincubation with 25 µM curcumin almost completely suppressed aggregation induced by both fibrillar Aβ40 and fibrillar Aβ42 (maximal aggregation %: curcumin + Aβ40: 12.4 ± 2.48; curcumin + Aβ42: 7.88 ± 3.18) (Figure 1A). To compare this effect of curcumin with that elicited upon stimulation with physiological platelet agonists, washed human platelets were preincubated with increasing concentrations of curcumin and then stimulated with a low dose of thrombin (0.04 U/mL). We found that curcumin dose-dependently reduces thrombin-induced aggregation (Figure 1B). Thrombin-induced aggregation was reduced by about 50% (IC 50 = 24.09 µM) by 25 µM curcumin; however, a complete inhibition of aggregation was observed only at doses of curcumin as high as 100 µM (Figure 1B). Curcumin also dose-dependently reduced platelet aggregation induced by the GPVI agonist convulxin (Figure 1C), and to a lesser extent by the thromboxane A2 (TxA2) analog U46619 (Figure 1D), but in these cases complete inhibition also required doses of curcumin much higher than 25 µM. Therefore, curcumin is far more potent in reducing platelet aggregation induced by amyloid peptides compared to classical standard agonists.

### 3.2. The Effect of Curcumin on α Granule Secretion Depends on the Platelet Agonist

**Figure 2:**
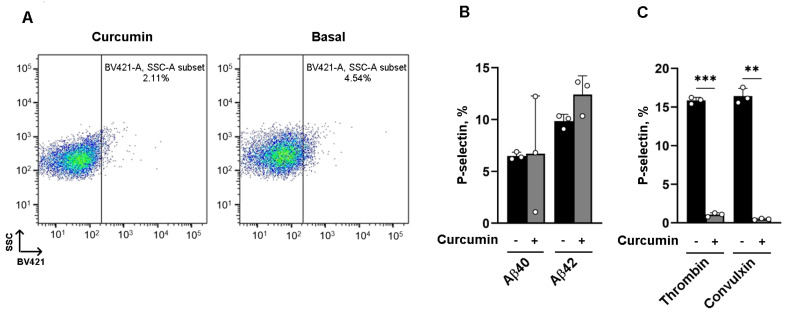
The effect of curcumin on α granule secretion depends on the platelet agonist: (**A**) Washed human platelets (1 × 10^8^ platelets/mL) labeled with BV421-conjugated anti-human CD62P (P-selectin) antibody were incubated with 25 µM curcumin alone (curcumin) or vehicle (basal). Representative dot plots are reported. (**B**) Histogram showing the mean ± SEM of the % of P-selectin-positive cells expressed on the plasma membrane in platelets preincubated with 25 µM curcumin and stimulated with 20 µM amyloid peptides Aβ40 and Aβ42 for 15 min. (**C**) Washed human platelets were preincubated with curcumin 25 µM for 10 min and stimulated with 0.1 U/mL thrombin or 100 ng/mL convulxin for 15 min at 37 °C. Data are expressed as the mean ± SEM of the % of P-selectin-positive cells; ** *p* < 0.01, *** *p* < 0.001.

We next investigated the effect of curcumin on α granule secretion induced by amyloid peptides, analyzed via flow cytometry on isolated human platelets. Platelets were preincubated with 25 µM curcumin or vehicle and stimulated with 20 µM fibrillar Aβ40 or Aβ42 for 10 min, and the exposure of P-selectin (CD62P) on the plasma membrane was measured as marker of α granule secretion. We observed that preincubation with curcumin had no effects on α granule release (Figure 2A), and that α granule release induced by Aβ42 was significantly stronger than that induced by Aβ40 (P-selectin positive cells %: Aβ40: 6.48 ± 0.208; curcumin + Aβ40 + curcumin: 6.71 ± 3.22; Aβ42: 9.84 ± 0.384; curcumin + Aβ42: 12.4 ± 1.04) (Figure 2B), despite Aβ42 being less potent in promoting platelet aggregation. These results suggest that aggregation and granule secretion induced by amyloid peptides are independently regulated by curcumin. In contrast, the hemostatic stimuli thrombin and convulxin promoted a much stronger α granule secretion, albeit this was severely impaired in the presence of curcumin (P-selectin positive cells %: thrombin: 15.8 ± 0.238; curcumin + thrombin: 1.05 ± 0.145; convulxin: 16.4 ± 0.586; curcumin + convulxin +: 0.482 ± 0.0592) (Figure 2C), suggesting that curcumin in not only differentially potent but also exerts differential effects depending on the nature of the platelet agonist. 

### 3.3. Curcumin Reduces the Phosphorylation of Selected Signaling Proteins

**Figure 3:**
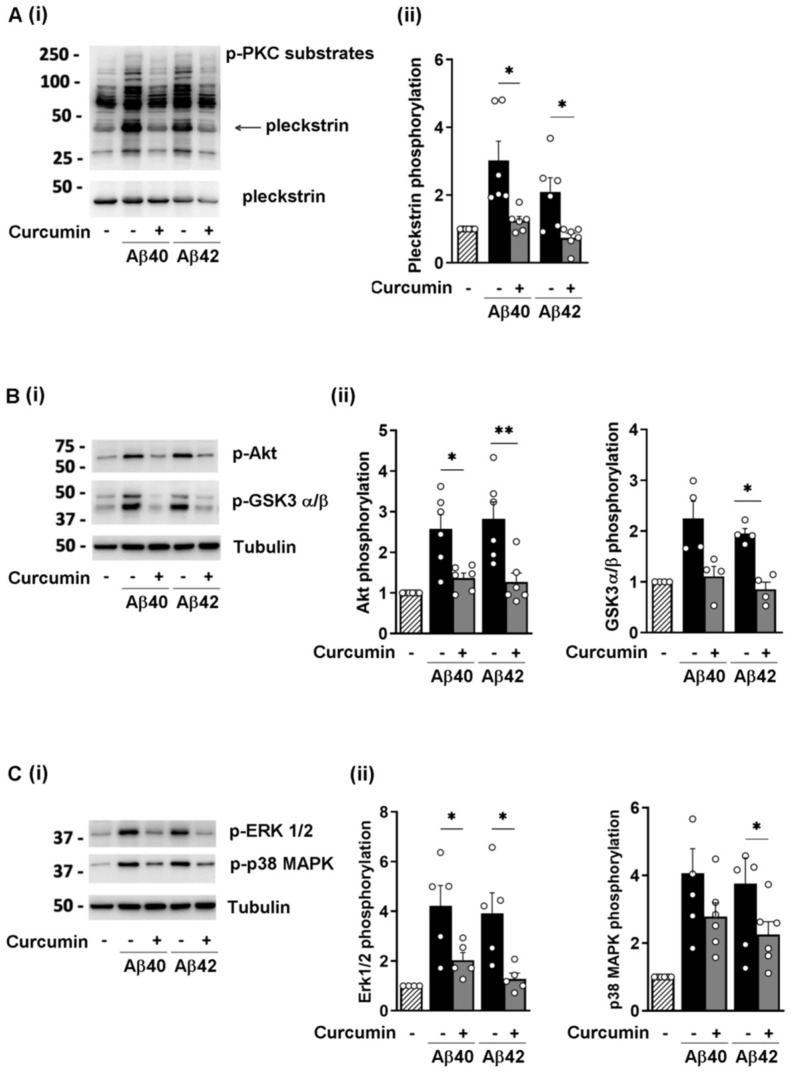
Curcumin suppressed the phosphorylation of signaling proteins in platelets stimulated with fibrillar amyloid peptides: Washed human platelets (1 × 10^9^ platelets/mL) were preincubated for 10 min with 25 µM curcumin and then stimulated with 10 µM fibrillar Aβ40 and Aβ42; 20 µL samples of platelet lysates were analyzed via immunoblotting with specific phospho-antibodies. Representative immunoblotting results are shown in (**i**). (**A**) Phosphorylation of PKC substrates; (**B**) phosphorylation of Akt (S473) and GSK3α/β (S21/9); (**C**) phosphorylation of ERK1/2 (T202/Y204) and p38 MAPK (T180/Y182). Pleckstrin and tubulin were used for equal loading controls. The histograms in (**ii**) show the mean ± SEM of 5 to 6 different experiments. Analysis of phosphorylation of selected proteins is compared to basal conditions, set as 1 in each experiment; * *p* < 0.05, ** *p* < 0.01.

To investigate the molecular mechanism mediating the inhibitory effect of curcumin on platelet activation induced by fibrillar Aβ40 and Aβ42, we analyzed the phosphorylation of selected kinases, including PKC, the PI3K downstream effectors Akt and GSK3, and MAPK, which had been shown to be activated by amyloid peptides [8]. We found that preincubation of washed platelets with 25 µM curcumin reduced the phosphorylation of PKC substrates to basal levels, in particular for p47 pleckstrin (Figure 3A), Akt and GSK3 α/β, and ERK1/2 and p38 MAPK (Figure 3B,C, respectively). Detection of total pleckstrin and tubulin was used as controls for equal sample loading, as shown in Figure 3. The levels of Akt, ERK2, and p38MAPK remained unchanged during the brief time of platelet incubation with curcumin and stimulation, as shown in Supplementary Figure S2.

**Figure 4:**
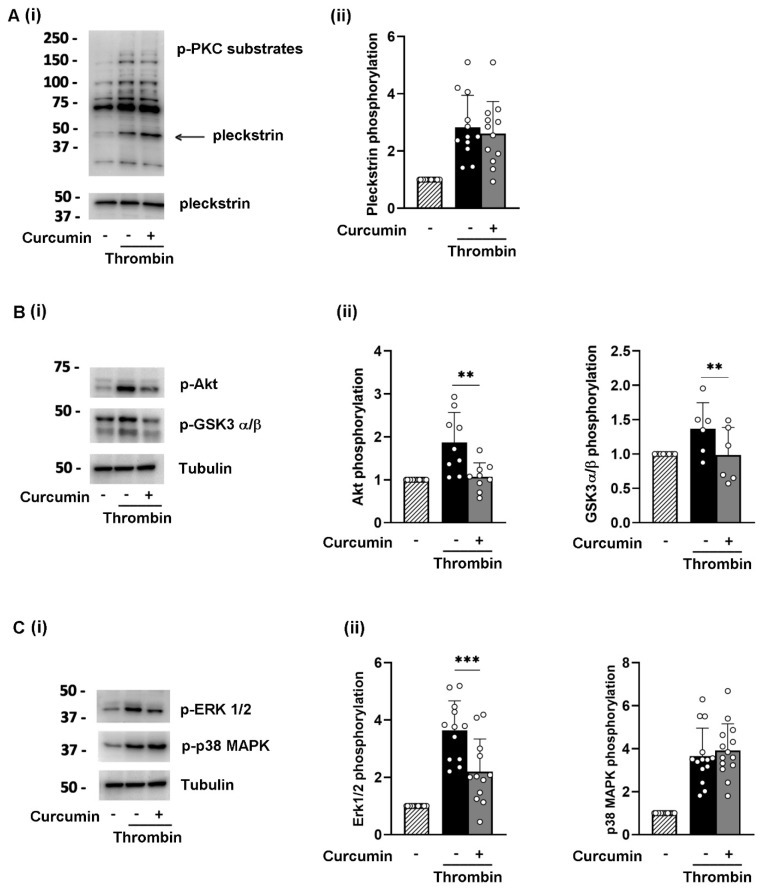
Curcumin reduces the phosphorylation of selected signaling proteins in platelets stimulated with thrombin: Washed human platelets (1 × 10^9^ platelets/mL) were preincubated for 10 min with 25 µM curcumin at 37 °C and stimulated with 0.04 U/mL thrombin for 5 min. Immunoblotting was performed to assess the phosphorylation of (**A**) PKC substrates, (**B**) Akt (S473) and GSK3α/β (S21/9), and (**C**) Erk1/2 (T202/Y204) and p38 MAPK (T180/Y182), and representative images are shown in (**i**). Pleckstrin and tubulin were used for equal loading controls. Quantification of phosphorylation of selected proteins is reported in the respective histogram (**ii**), where phosphorylation in non-stimulated conditions was set as 1. Data are the mean ± SEM of 6 to 12 independent experiments; ** *p* < 0.01, *** *p* < 0.001.

Surprisingly, 25 µM curcumin had a minimal effect on thrombin-induced protein phosphorylation. As shown in Figure 4, thrombin-induced PKC and p38MAPK phosphorylation was not affected by curcumin, while its effects on Akt, GSK3α/β, and ERK, albeit statistically significant, were very modest. Detection of pleckstrin and tubulin was used for equal loading controls. Incubation with curcumin and platelet stimulation did not change the total levels of Akt, ERK2, and p38MAPK, as reported in Supplementary Figure S2. Moreover, when the platelets were stimulated with convulxin, the effect of curcumin on protein phosphorylation was comparable to that observed upon thrombin stimulation (Supplementary Figure S1).

### 3.4. Curcumin Inhibits Intracellular ROS Formation

**Figure 5:**
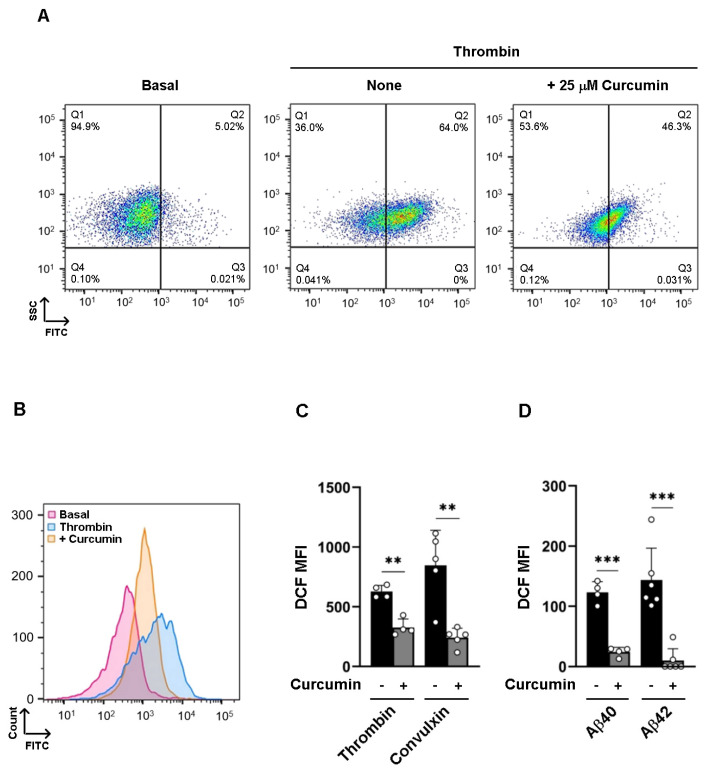
Curcumin inhibits ROS formation: Washed human platelets (1 × 10^8^ platelets/mL) were preloaded with 10 µM H_2_DCF-DA for 20 min in the dark, preincubated with vehicle (none) or 25 µM curcumin (curcumin), and stimulated with 0.1 U/mL thrombin. Representative (**A**) dot plots and (**B**) curves are shown. Analysis of DCF mean fluorescence intensity (DCF MFI) measured in platelets stimulated with (**C**) 0.1 U/mL thrombin and 100 ng/mL convulxin, or (**D**) 25 µM fibrillar Aβ40 and 25 µM fibrillar Aβ42, for 15 min at 37 °C. Data are the mean ± SEM of 4 to 6 different experiments; ** *p* < 0.01, *** *p* < 0.001.

Curcumin displays antioxidant properties that are crucial in maintaining the redox balance of the cells but also contribute to cellular signaling. Amyloid peptides, as well as physiological agonists, are known to promote NOX-dependent ROS formation [8]. To compare the effects of curcumin on amyloid peptides and thrombin- or convulxin-induced ROS production, platelets were loaded with the cell-permeant probe H2DCFDA, stimulated with agonists in the presence of curcumin, and analyzed by flow cytometry. Stimulation of platelets with 0.04 U/mL thrombin or 100 ng/mL convulxin promoted rapid and strong ROS production, which was significantly reduced but not completely suppressed by preincubation with curcumin (Figure 5A–C). By contrast, stimulation of platelets with the fibrillar amyloid peptides Aβ40 or Aβ42 resulted in a lower and slower ROS production, albeit this was totally suppressed by preincubation with curcumin (Figure 5D).

## 4. Discussion

Curcumin has pleiotropic effects with anti-inflammatory, antioxidant, and anti-amyloidogenic properties [10,11,12,13,17]. Curcumin and curcuminoids show antithrombotic and cardiovascular-protective effects that result from their ability to reduce platelet activation and aggregation, to prolong the clotting time, and to diminish fibrin deposition [22].

Many efforts have been made to understand the anti-platelet effect of curcumin at a molecular level; however, the investigations have been hampered by the fact that different sources of curcumin (crude curcumin, curcuminoids, turmeric extract, curcumin derivatives) and different doses have been used in the past [16,17,23].

In the present study, we used commercially available, ultrapure curcumin to investigate its effects on platelet aggregation, activation, and ROS formation induced by pathological Aβ peptides compared to those elicited upon platelet activation by physiological agonists. While it is well known that platelet activation induced by hemostatic physiological stimuli is crucial in the regulation of hemostasis and thrombosis, the role of amyloid peptides as platelet agonists in the bloodstream is still debated. Amyloid peptides are produced and stored at low levels in circulating platelets (Aβ40: 83 ng/g, Aβ42: 1.7 ng/g in quiescent platelets [20]) and are released during platelet activation [24]. Platelet-derived amyloid peptides reinforce platelet activation [5,6], fibrin clot stability [25], and thrombus formation [26]. Recently, it has been postulated that Aβ peptides also have antimicrobial properties, participate in innate immunity by entrapping and killing bacteria, and may be released as a primary defense to infection [27]. Here, we show that platelet activation and aggregation induced by the fibrillar amyloid peptides Aβ40 and Aβ42 are completely suppressed by doses of curcumin that display only a limited effect on the same responses triggered by stimulation with physiological agonists. This is of particular interest in the circulation, where amyloid peptides are likely to contribute to a low-grade pro-thrombotic and pro-inflammatory state. Such pathological states, which are observed in AD patients and lead to cardiovascular complications, can be attenuated by curcumin treatment.

It has been demonstrated that curcumin inhibits the aggregation of Aβ peptides and is able to disintegrate preformed Aβ fibrils and amyloid plaques [28]. In our experimental model, however, the ability of curcumin to inhibit platelet aggregation does not rely on the modulation of amyloid fibrillation, because the Aβ40 and Aβ42 fibrils were preformed for 24 h before addition to curcumin-treated platelets, and the success of the fibrillation process was confirmed by a Thioflavin T assay [8]. Moreover, platelet stimulation with fibrillar Aβ peptides was performed for 5 min, a time not sufficient to allow for the disaggregation of fibrils by added curcumin [29]. Therefore, curcumin affects Aβ peptide-induced platelet activation by targeting the platelet response rather than the structure of platelet agonists.

Interestingly, the dose of curcumin that completely suppresses platelet aggregation induced by fibrillar amyloid peptides only partially reduces platelet activation and aggregation induced by classical physiological agonists such thrombin, the GPVI agonist convulxin, and the thromboxaneA2 analog U46619. The different potency of curcumin in modulating platelet responses to these different agonists may be due to the fact that hemostatic or pathological stimuli can activate specific signaling pathways that are differently sensitive to the action of curcumin. This different modulation could have clinical applications in the treatment of CVD observed in AD patients.

Fibrillar amyloid peptides are known to bind to the GPIb-IX-V complex and CD36, and to signal through PKC, MAPK, and Akt activation as well as through ROS formation to induce full platelet activation [8]. Interestingly, curcumin completely inhibits PKC, PI3K, and MAPK induced by amyloid peptides. This may explain the almost complete inhibition of platelet aggregation. We also found that curcumin does not affect platelet secretion induced by amyloid peptides, suggesting that this platelet response is minimally regulated by the signaling proteins investigated. This is in line with the observation that when platelets are stimulated with physiological agonists such as thrombin, platelet secretion is strongly inhibited by curcumin, although the PKC, MAPK, and PI3K pathways are only marginally affected. It has been postulated that curcumin also increases the levels of the cyclic nucleotides cAMP and cGMP [14], which are known to inhibit activation. The possibility that PKA and PKG activation may contribute to the inhibition of Aβ peptide-induced platelet activation cannot be excluded and deserve further investigations.

We have also demonstrated that curcumin affects Aβ-induced platelet activation through antioxidant properties. In platelets, ROS are primarily produced by the action of NOX and by mitochondrial respiration, and they are known to modulate platelet functions. Amyloid peptides produce low amounts of ROS, with slower kinetics compared to the classical agonists thrombin and convulxin. However, ROS generation supports platelet aggregation, as ROS scavengers or inhibition of NOX can result in reduced aggregation in platelets stimulated with amyloid peptides [8]. Curcumin suppresses ROS production induced by fibrillar Aβ40 and Aβ42, and it strongly inhibits thrombin and convulxin, highlighting the pivotal antioxidant role of curcumin in platelets. The mechanism by which curcumin blocks ROS production in platelets is not known, but we can speculate that curcumin negatively regulates phosphorylation of NOX by inhibiting PKC activation. This hypothesis, however, needs further investigation. Moreover, it should also be considered that curcumin acts as an ROS scavenger thanks to its two phenolic sites [30] and may increase the action of antioxidant enzymes such as superoxide dismutase and catalase [31]. All of these properties may contribute to prevention of ROS accumulation in Aβ-stimulated platelets.

The effect of curcumin on the progression of AD in humans is barely known. Curcumin, in fact, is a hydrophobic polyphenol and, therefore, has a restricted ability to cross the blood–brain barrier (BBB), limiting its therapeutic potential in the brain [32]. Nevertheless, some studies suggest that curcumin treatment may be beneficial in the treatment of neurological disorders [33] such as AD, when the permeability of the BBB is compromised. For instance, in a well-studied mouse model of AD (APP/PS1 mice), curcumin reduced the formation of amyloid protein plaques as well as oxidative stress and ameliorated cognitive functions by inhibiting the HMGB1-RAGE/TLR4-NF-kappaB signaling pathway [34].

Other important issues that limit the therapeutic use of curcumin include its extremely poor pharmacokinetic/pharmacodynamic properties, its chemical instability, and its toxic profile evidenced under specific experimental settings [35] and associated with serious hepatocellular damage. The development of novel delivery systems based on nanotechnology, novel administration methods, and alternative formulations have all contributed to the resolution of important pharmaceutical problems associated with curcumin’s pharmacokinetics, enhancing its therapeutic efficacy and raising new hopes for the future clinical use of this natural compound [36]. Indeed, it has been established that entrapment in poly D,L-lactic-co-glycolic acid nanoparticles is appropriate for delivering curcumin to target tissues, as well as for enhancing its activity and boosting an early cell-mediated immune response [37].

## 5. Conclusions

In conclusion, our results demonstrate that curcumin is an efficient inhibitor of platelet activation induced by classical hemostatic agonists in vitro, but it displays a stronger and more pronounced effect when platelets are challenged by fibrillar amyloid peptides. Despite the limitations of the use of curcumin due to its poor bioavailability in vivo, this study suggests that curcumin may be of particular efficacy and selectivity in reducing cardiovascular complications observed in AD patients. Further investigations in transgenic mouse models of AD are needed to confirm the antithrombotic potential of curcumin in vivo. Moreover, its applicability in mouse models and in humans could benefit from the development of novel curcumin formulations, such as micelle and phospholipid complexes, liposomal curcumin, and delivery systems based on nanoparticles, which have been evaluated to counteract pharmaceutical problems associated with curcumin’s pharmacokinetics [36].

## Data Availability

Data are contained within the article and Supplementary Materials.

## Supplementary Materials

The following supporting information can be downloaded at https://www.mdpi.com/article/10.3390/nu16244419/s1: Figure S1: Curcumin reduces the phosphorylation of selected signaling proteins in platelets stimulated with convulxin; Figure S2: Expression of Akt, ERK2 and p38MAPK in amyloid peptides-and thrombi-stimulated platelets in the presence of curcumin.
