# Supplementary material for: Curcumin Modulates Platelet Activation and ROS Production Induced by Amyloid Peptides: New Perspectives in Attenuating Prothrombotic Risk in Alzheimer’s Disease Patients
Source: Nutrients. 2024 Dec 23;16(24):4419. doi: 10.3390/nu16244419 (PMC11678805; doi:10.3390/nu16244419)
Supplement: Supplementary file 1 [file nutrients-16-04419-s001.zip › nutrients-3346056-supplementary.pdf]

Supplemental Figure S1

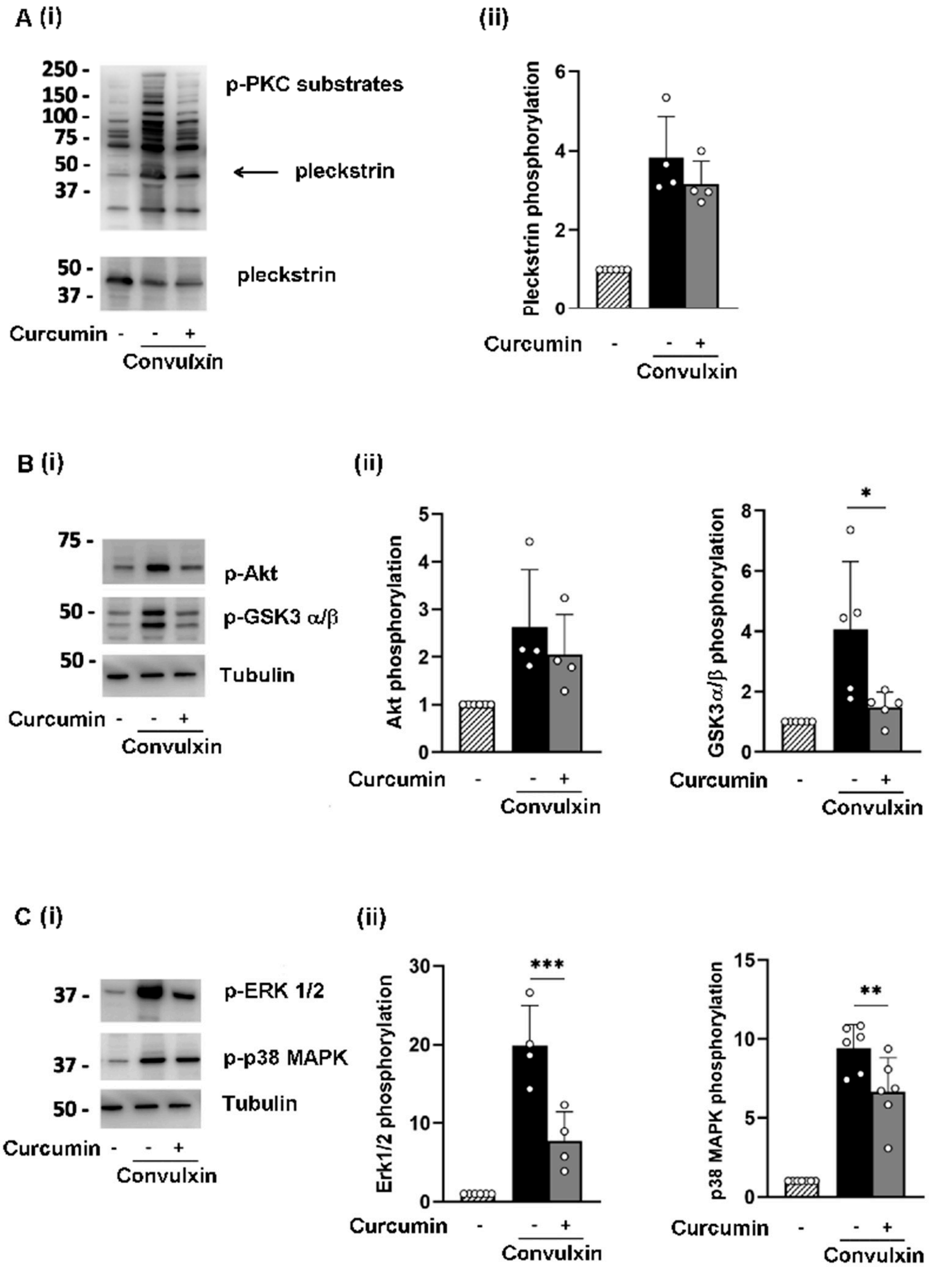

Curcumin reduces the phosphorylation of selected signaling proteins in platelets stimulated with convulxin. Washed human platelets (1 x 10^9 platelets/mL) were preincubated for 10 minutes with curcumin 25 μM at 37 °C and then stimulated with convulxin 100 ng/ml for 5 minutes. Immunoblotting (i) was performed to assess the phosphorylation of PKC-substrates (A), Akt (Ser^{473}) and GSK-3 α/β (Ser^{21}/Ser^{9}) (B), Erk1/2 (Thr^{202}/Tyr^{204}) and p38 MAPK (Thr^{180}/Tyr^{182}) (C). Pleckstrin or tubulin were used for equal loading control as indicated. Quantification of phosphorylation of selected proteins is reported in the respective histogram (ii), where phosphorylation in non-stimulated condition was set as 1. Data are the mean ± SEM of 6-12 independent experiments. ** $p < 0.01$, and *** $p < 0.001$.

Supplemental Figure S2

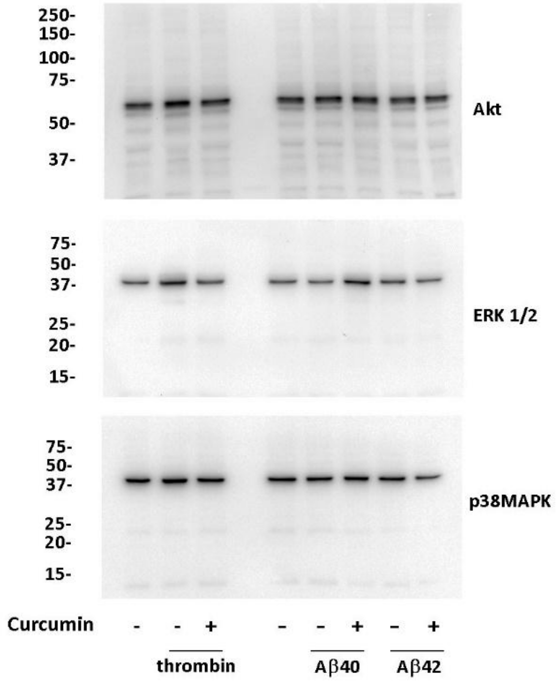

Expression of Akt, ERK1/2 and p38MAPK in amyloid peptides-and thrombin-stimulated platelets in the presence of curcumin. Washed human platelets (1 x 10^9 platelets/mL) were preincubated for 10 minutes with curcumin 25 μM at 37 °C and then stimulated with fibrillar 10 μM amyloid peptides Aβ40 and Aβ42, or with thrombin 0.04U/mL for 5 minutes as reported. 20 μl of platelet lysates were analysed in immunoblotting for protein expression by using antibodies against Akt, ERK1/2 and p38MAPK. Representative immunoblotting are shown and standard molecular weight are reported on the left.
